# ALK (anaplastic lymphoma kinase, CD246)-specific CARs: new immunotherapeutic agents for the treatment of pediatric solid tumors

**DOI:** 10.1186/2051-1426-1-S1-P27

**Published:** 2013-11-07

**Authors:** Rimas J Orentas, Paola Lopomo, William Babbitt, Marc Vigny, Crystal L Mackall

**Affiliations:** 1 Pediatric Oncology Branch, CCR, NCI, NIH, Bethesda, MD, USA; 2 Institut du Fer à Moulin, INSERM/UPMC, Paris, France

## 

The identification of unique cell-surface proteins expressed on tumor cells, yet not expressed on normal tissues, has been challenging for pediatric malignancies. The cell surface tyrosine kinase ALK (CD246, anaplastic lymphoma kinase) is a promising target for neuroblastoma in that it is expressed in either native, mutated, or over-expressed forms on the plasma membrane surface. We identified antibodies that bind to ALK, sequenced their variable regions, and used this sequence information to construct chimeric antigen receptors (CARs). Primary human T lymphocytes were then transduced with retroviral gene vectors expressing a series of ALK-specific CARs, that included different structural and signaling motifs. Transduced T cells demonstrated ALK-specific cytolytic activity against ALK-expressing tumors and produced Th1 cytokines upon culture in the presence of tumor. In exploring different iterations of CAR protein domain structure we found that the scFv domains created from the heavy and light variable domains of ALK-specific immunoglobulin could be interchanged with respect to their orientation in the context of CAR tertiary protein structure. Moreover, ALK-specific scFv functioned whether expressed in a short format, that is as a single domain proximal to the T cell membrane, or in a long format, that is extended away from the plasma membrane using an IgG1-derived spacer domain composed of CH2 and CH3. Using a xenogeneic NSG mouse model for neuroblastoma, human ALK-specific CAR-expressing T cells were found eradicate ALK-positive tumor, when IL-7 was included to support T cell persistence. These data argue for the continued evaluation of ALK-specific CARs in pre-clinical studies.

